# Fluorescent Polystyrene Films for the Detection of Volatile Organic Compounds Using the Twisted Intramolecular Charge Transfer Mechanism †

**DOI:** 10.3390/molecules22081306

**Published:** 2017-08-06

**Authors:** Mirko Borelli, Giuseppe Iasilli, Pierpaolo Minei, Andrea Pucci

**Affiliations:** 1 Dipartimento di Chimica e Chimica Industriale, Università di Pisa, Via G. Moruzzi 13, 56124 Pisa, Italy; mirkobore@live.it (M.B.); giuseppe.iasilli@gmail.com (G.I.); Pierminei@gmail.com (P.M.); 2 INSTM, UdR Pisa, Via G. Moruzzi 13, 56124 Pisa, Italy

**Keywords:** fluorescent molecular rotors, polymer films, VOC, vapochromism

## Abstract

Thin films of styrene copolymers containing fluorescent molecular rotors were demonstrated to be strongly sensitive to volatile organic compounds (VOCs). Styrene copolymers of 2-[4-vinyl(1,1′-biphenyl)-4′-yl]-cyanovinyljulolidine (JCBF) were prepared with different P(STY-*co*-JCBF)(m) compositions (m% = 0.10–1.00) and molecular weights of about 12,000 g/mol. Methanol solutions of JCBF were not emissive due to the formation of the typical twisted intramolecular charge transfer (TICT) state at low viscosity regime, which formation was effectively hampered by adding progressive amounts of glycerol. The sensing performances of the spin-coated copolymer films (thickness of about 4 µm) demonstrated significant vapochromism when exposed to VOCs characterized by high vapour pressure and favourable interaction with the polymer matrix such as tetrahydrofurane (THF), CHCl_3_ and CH_2_Cl_2_. The vapochromic response was also reversible and reproducible after successive exposure cycles, whereas the fluorescence variation scaled linearly with VOC concentration, thus suggesting future applications as VOC optical sensors.

## 1. Introduction

Nowadays, chromogenic fluorescent (fluorogenic) materials are being effectively used for the preparation of devices showing optical features sensitive to different external stimuli [1,2,3,4,5,6,7,8]. The fluorescent properties generally depend on different molecular parameters, such as structural flexibility, electron donor or acceptor moieties in addition to an extended conjugation. Nevertheless, self-assembly of fluorophore molecules usually suffer of emission quenching phenomena (aggregation-caused quenching (ACQ)), which strongly restricts the number of accessible fluorophores for practical applications. In this context, a revolutionary class of luminophores demonstrating the fluorescence development with aggregation has drawn great attention since their discovery sixteen years ago by Ben Zhong Tang [9,10,11,12]. The effect, called aggregation induced emission (AIE), arises from the restriction of fluorophore intramolecular motions (RIM), which promotes bright luminescence in the aggregate and solid state [5,7]. By enabling light emission in the practically useful solid state, AIE fluorophores demonstrate to show a striking impact on energy, optoelectronics, life science and environment [9,13,14,15,16,17,18]. Notably, for AIE systems with donor-acceptor structure, the emission quenching is often addressed to the formation of a non-emissive twisted intramolecular charge transfer (TICT) state that occurs in solution, while in aggregates or in viscous media, transition from locally excited (LE) state to TICT is inhibited [10]. Those molecules, called also fluorescent molecular rotors (FMRs), have become rather popular in the last 5–10 years thanks to their easy applicability as non-mechanical viscosity sensors, tools for protein characterization, and local microviscosity imaging [19,20,21,22]. Viscosity measurements by means of FMRs require shorter measurements times and smaller amounts of samples. Moreover, since the fluorescence viscometry does not apply shear to the sample, it is more practical for biofluids, which have apparent non-Newtonian properties. FMRs in solution has been explored since years, and examples of their use in combination with polymers have been efficiently reported for the detection of volatile organic compounds (VOCs) [22,23,24,25]. The detection of VOCs is an important issue considering that they are continuously released into the environment by different sources such as industrial processes, transportation, agriculture, as well as indoor applications, and some of them have adverse effects on human health [26,27]. VOCs are usually characterized by low boiling point and high vapour pressure at standard conditions, thus they may rapidly fill an enclosed environment. Because of their toxic nature, in many states there are regulations setting a limit to VOCs emission. Furthermore, current pressing concerns in global security have stimulated the development of new fluorescent materials, with various sensing mechanism, aimed at detecting chemicals in the vapour phase. For example, the vapochromism of julolidine-based FMRs were explored in polystyrene (PS) films [28]. The exposure of FMR/PS films to well-interacting VOCs induced plasticization of the supporting polymer matrix, thus favouring a striking drop of FMR fluorescence due to the favoured relaxation from the non-emissive TICT state. Nevertheless, efforts must be still pursued to confer the fastest response under VOCs exposure. For example, it is expected that covalent binding of FMR moieties to the macromolecular chains should provide the fastest response to minimal amounts of VOCs. Notably, the amount of FMR covalently linked to the polymer can be easily modulated by adjusting the content of the labelled co-monomer in the feed [3,23]. This procedure would also ensure a more homogeneous distribution of the active FMR units within the macromolecular chains, without promoting the formation of stacked supramolecular structures potentially useless for the vapochromic response. In the present work, styrene copolymers of 2-[4-vinyl(1,1′-biphenyl)-4′-yl]-cyanovinyljulolidine (JCBF) were prepared with different P(STY-*co*-JCBF)(m) compositions with m% = 0.10–1.00. Copolymer thin films were obtained by spin-coating on glass plate surfaces, and their sensing performances towards different VOCs were investigated at room temperature.

## 2. Results and Discussion

### 2.1. Synthesis of JCBF

**Scheme 1:**
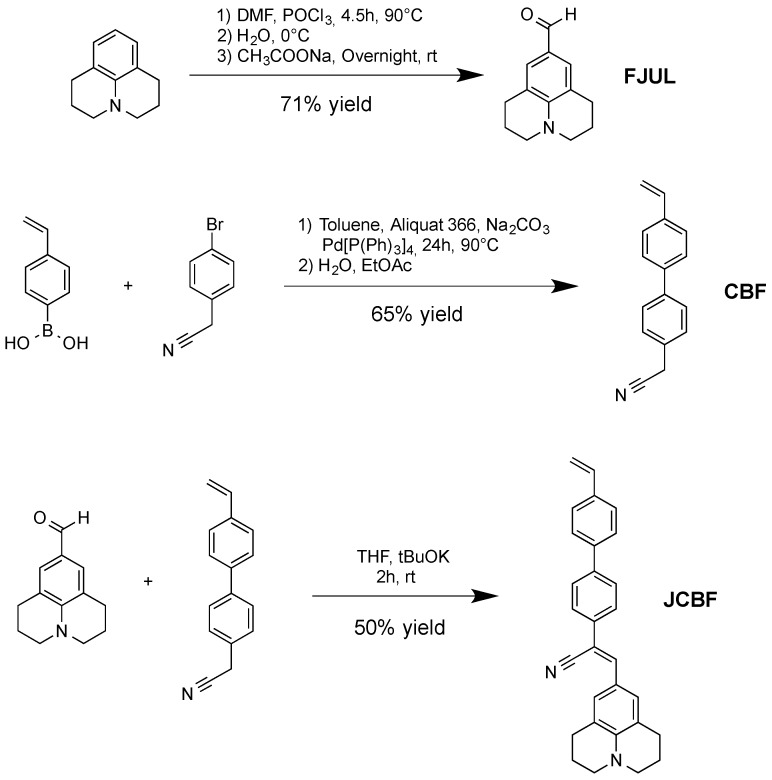
Synthesis of the 2-[4-vinyl(1,1′-biphenyl)-4′-yl]-cyanovinyljulolidine (JCBF) monomer. DMF: dimethylformamide; rt: room temperature; THF: tetrahydrofurane; FJUL: 9-formyljulolidine; CBF: of 2-[4-vinyl(1,1′-biphenyl)-4′-yl]-acetonitrile.

The synthesis of the 2-[4-vinyl(1,1′-biphenyl)-4′-yl]-cyanovinyljulolidine (JCBF, Scheme 1) and its inclusion into styrene polymers by conventional radical polymerization have never been reported before. JCBF should act as a viscosity-sensitive FMR unit in glassy PS environment owing to the presence of the cyanovinyljulolidine moiety. Notably, the FMR unit is linked to the vinylbiphenyl moiety that is more chemically related to the styrene repeating units than the methacrylate functions used earlier [25].

**Scheme 2:**
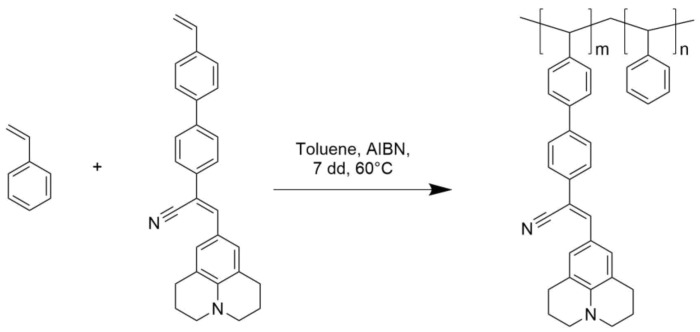
Synthesis of the P(STY-co-JCBF) copolymers. AIBN: Azobisisobutyronitrile. dd: days.

Julolidine was formylated with phosphorus oxychloride and dimethylformamide to afford 9-formyljulolidine (FJUL). The 2-(4′-vinyl-[1,1′-biphenyl]-4-yl)acetonitrile was obtained via cross-coupling between 2-(4-bromophenyl)acetonitrile and 4-vinylphenylboronic acid as reported in literature [29]. The product was then reacted with FJUL, thus yielding after purification the desired target monomer JCBF with a yield of 50%. The appropriate amount of JCBF was then copolymerized with styrene through radical polymerization with Azobisisobutyronitrile (AIBN) for one week to afford P(STY-co-JCBF)(m) copolymers, with m% = 0.10–1.00 (Scheme 2).

Copolymer compositions, molecular weights and thermal behaviour are reported in Table 1.

A long polymerization time (7 days) was required to maximize JCBF conversion. The JCBF monomer was barely inclined to propagate, possibly due to an effective stabilization of the radical moiety by its chromophoric unit.

Four distinct copolymers were prepared and containing progressive amounts of JCBF, i.e., from 0.10 to 1.04 mol %, as determined by ^1^H-NMR and UV-Vis experiments, that is supposing negligible variation of the molar extinction coefficient of JCBF after polymerization. No evidence of JCBF content on the copolymers molecular weight and thermal properties is present. Notably, P(STY-co-JCBF) copolymers display a glass transition temperature in between 100 and 110 °C, and a degradation onset above 415 °C.

### 2.2. Optical Properties of JCBF Chromophore in Solution

**Figure 1:**
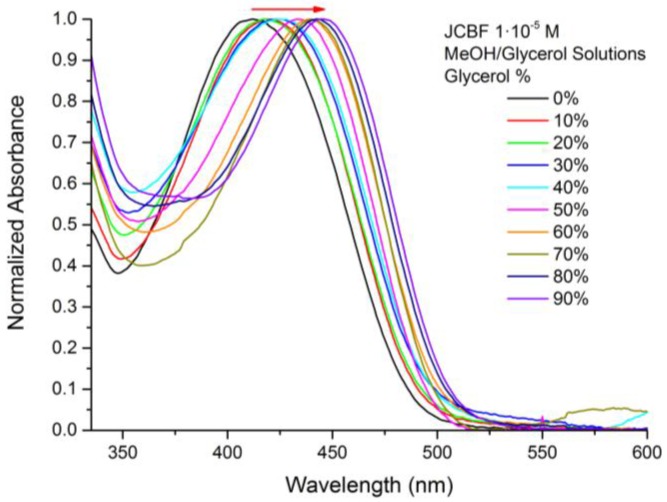
UV-Vis absorption spectra of 1 × 10^−5^ M JCBF in methanol/glycerol solutions with different glycerol volume contents.

JCBF chloroform solution showed absorption maximum around 407 nm (*ε* = 21,500 M^−1^ cm^−1^), with emission maximum at about 500 nm with a negligible quantum yield (*Φ_f_*) of 2.5∙× 10^−3^ due to the favored formation of the typical radiationless TICT excited state (Figure S1) [30,31]. Similar absorption (*A_max_* = 407 nm) and emission (*Em_max_* = 500 nm) were recorded in methanol solutions as well as for tetrahydrofurane (THF) (*A_max_* = 407 nm, *Em_max_* = 505 nm) and toluene (*A_max_* = 403 nm, *Em_max_* = 494 nm). Surprisingly, a strongly different behavior was observed when glycerol was added to methanol solutions (Figure 1 and Table 2).

**Figure 2:**
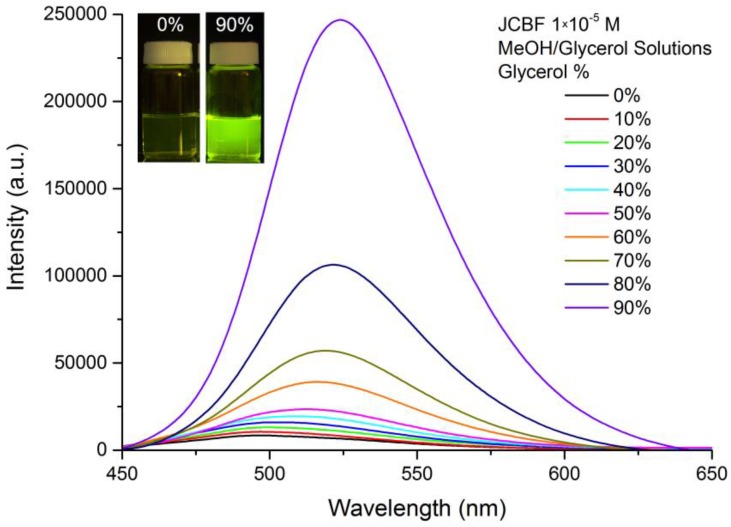
Fluorescence (λ_exc._ = 410 nm) of 1 × 10^−5^ M JCBF in methanol/glycerol solutions with different glycerol volume contents. In the inset picture, JCBF solutions were excited with a Dark Reader 46B transilluminator (~450 nm, Clare Chemical Research, Dolores, CO, USA).

JCBF exhibited an apparent solvatochromism (55 nm in absorption and 20 nm in emission) when glycerol was added to methanol solutions (Figure 1 and Figure 2, Table 2). The red-shift in the absorption spectrum with increasing polarity is not typical in FMRs, which usually exploit a solvent-independent absorption features [24,36]. To the best of our knowledge, Karpenko et al. have reported one of the few examples of FMR with evident solvatochromism also in absorption, even if at lower extent than JCBF. [37] This feature was addressed to the presence of a significant dipole moment in the ground state, which further increases on electronic excitation to the Frank-Condon state. Nevertheless, all these findings do not completely explain the effect of glycerol on JCBF methanol solution, and more accurate investigations are required. Although meaningful, we deferred the study to a future work.

As instead expected, in the excited state the molecule undergoes Intramolecular Charge Transfer (ICT) as it is favored in fluorophores bearing electron donor and acceptor moieties, thus showing fluorescent solvatochromism. More than that, the significant viscosity-dependent fluorescence of the JCBF monomer was noteworthy (Figure 2 and Table 2).

Notably, JCBF underwent a strong increase in quantum yield (about 30 times higher) when dissolved in viscous environments like glycerol-containing solutions (viscosity *η* = 630 mPas at 20 °C for methanol/glycerol 10:90 *v*/*v* mixture, as compared to 0.6 mPas for pure methanol). According to the FMR behaviour the molecular internal rotation of the molecule is hampered in viscous media, thus promoting the emission from the LE and, in turn, the increase in quantum yield. As expected, JCBF solutions ensued the typical Förster-Hoffmann behaviour [38] (Equation (1) and Figure S2), which relates the (double logarithmic) quantum yield with viscosity:

*logΦ_f_* = *C* + *x·logη* (1)

where C and x are constants. η was predicted by the Grunberg-Nissan simplified additive rule in Equation (2) [33]:

(2) $$\eta_{mix}=\phi_{1}\eta_{1}+\phi_{2}\eta_{2}$$

where the subscripts 1 and 2 are referred to solvent 1 and 2, respectively, $\phi_{i}$ are the volume fractions, and $\eta_{i}$ are the viscosities of pure substances [33]. The *x* parameter, defined as the viscosity sensitivity of the FMR, was found to be 0.56, which is comparable with the highest values reported for similar systems, thus making JCBF suitable as microviscosity probe of environments.

The JCBF chromophore did not experience any significant variation of its optical features when incorporated in the PS backbone. Notably, P(STY-co-JCBF) dissolved in chloroform displayed absorption peaked at 410 nm with emission pointed at about 500 nm with a negligible *Φ_f_* of 2.5 × 10^−3^ (Figure S3). Moreover, the progressive increase of the JCBF content in the PS backbone did not alter the position of the absorption and emission maxima peaks (Figure S4). Being P(STY-co-JCBF) polymers insoluble in methanol, no comparison with JCBF methanol solution was reported.

### 2.3. Vapochromism of P(STY-co-JCBF)(m) Thin Films

Well homogeneous P(STY-co-JCBF)(m) thin polymer films (4 µm) were obtained by spin-coating chloroform solutions on 2.4 × 2.4 cm cleaned glass cover slides. Absorption features appeared very similar to those gathered in solution and with absorbance maxima in agreement with the JCBF content (Figure S5a). As far as the emission is concerned, strong fluorescence was gathered from the solid films being the highly viscous and glassy (*T_g_* of about 100–110 °C, Table 1) polymer matrix favour light emission from the Locally Excited (LE) states of the covalently bonded JCBF moieties. The red-shift of the emission band with JCBF content (Figure S5b) could be possibly addressed to auto-absorption phenomena (inner-filter effect) [39].

**Figure 3:**
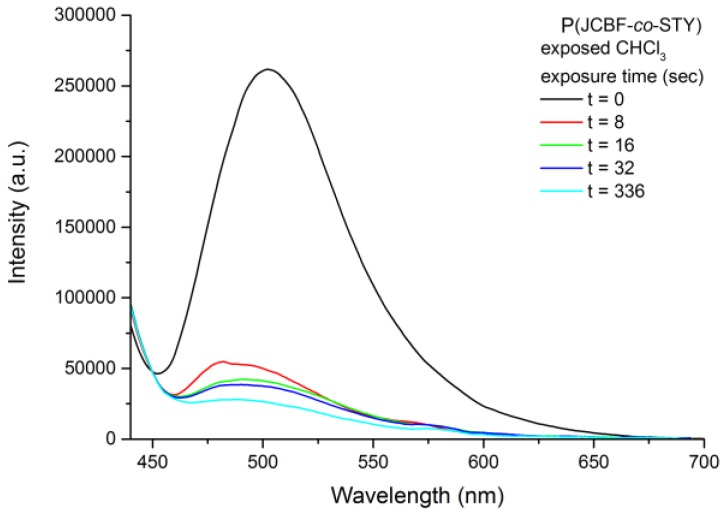
Progressive changes in the fluorescence emission (*λ_exc._* = 410 nm) of P(STY-co-JCBF)(0.34) film as a function of the exposure to chloroform vapours. The spectra were collected for a total time of 5–6 min.

The emission behaviour of P(STY-co-JCBF)(m) thin films was then studied by exposing them to different kinds of volatile organic solvents. An example of the fluorescence emission variation on exposure time to chloroform vapours is reported in Figure 3. It is worth noting that the P(STY-co-JCBF)(0.34) films experienced a significant variation in emission intensity just during the first minutes of VOC exposure (Figure 3).

The emission intensity dropped by about 80% after 8 s of exposure. This extremely sensitive solvent dependence, never achieved previously by our research group [22,24,25,28,40], agrees well with the characteristics of JCBF. Notably, the progressive VOC adsorption and successive absorption by the polymer matrix triggers the reorganization energy of the JCBF excited transition state from the LE to the non-emissive TICT. As already known, chloroform is a good solvent for PS (Table 3), whose empty channels and holes of molecular dimensions are filled by solvent vapours. Their progressive diffusion and the resulting swelling of the polymer causes rapid local microviscosity decrease, thus enabling fluorescence quenching. Notably, after a few minutes, the swelled polymer reached an equilibrium and its emission did not change any longer for prolonged exposure times.

**Figure 4:**
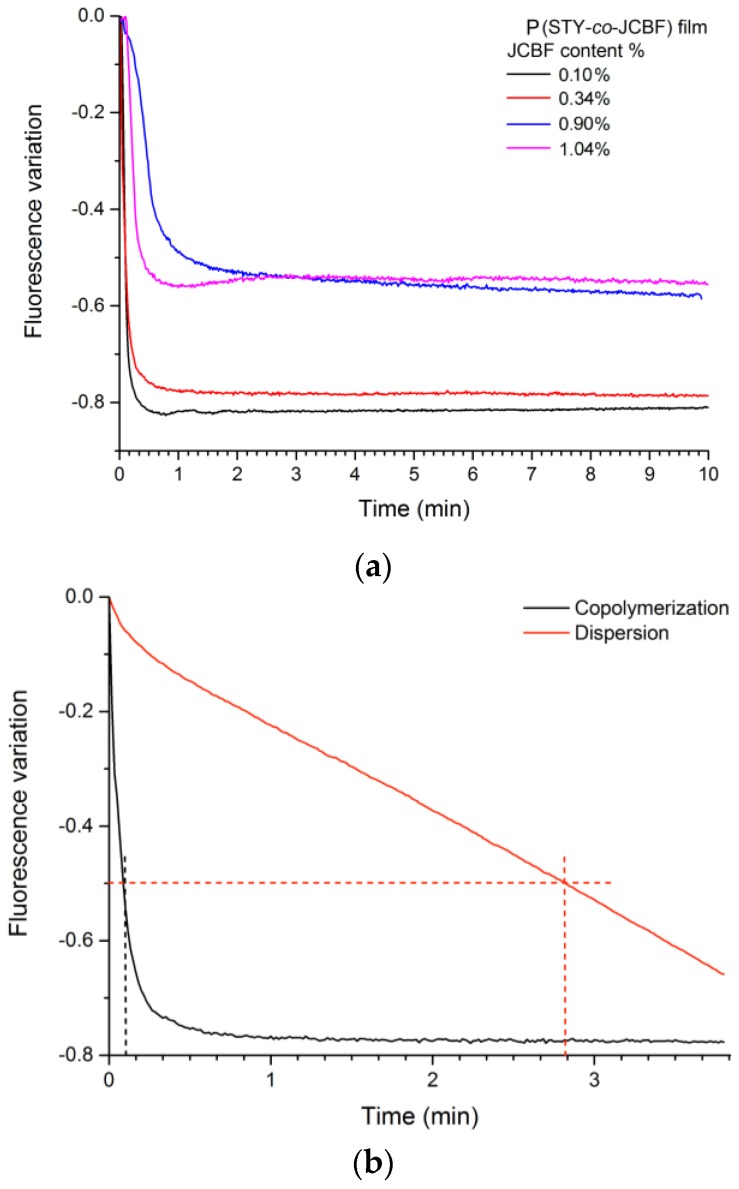
(**a**) Variation of the fluorescence maximum intensity (*λ_exc._* = 410 nm) with exposure time to chloroform vapours for all P(STY-co-JCBF)(m) films. Fluorescence was collected for a total time of 10 min in each experiment. (**b**) Comparison between P(STY-co-JCBF)(0.34) and the most sensitive julolidine-based Fluorescent Molecular Rotors (FMR) dispersed in polystyrene (PS) [28] towards chloroform vapours.

The monitoring of the fluorescence peak intensity variation as a function of VOCs exposure time (Figure 4) furnishes a better perception of the vapochromic response. All P(STY-co-JCBF)(m) thin films were monitored and exhibited a similar behaviour upon exposure to chloroform vapours. Fluorescence decreasing resulted faster for polymer films with the lowest JCBF content (i.e., P(STY-co-JCBF)(0.10) and P(STY-co-JCBF)(0.34)) and reached a plateau after less than 60 s (Figure 4). This phenomenon was addressed to the fact that low amounts of emitting species are expected to be readily solvated by the absorbed solvent and, therefore, to provide the greatest variation in emission during the early stages of VOCs exposure. Conversely, at higher fluorophore content, possible cluster formation due to π-π staking interactions might occur and retard the complete solvation of the single chromophoric units, which, in turn causes a substantial delay of the fluorescent quenching.

More specifically, the P(STY-co-JCBF)(0.10) film lost 50% of its pristine emission upon less than 10 s of exposure to chloroform vapours. By contrast, the most efficient julolidine-based FMRs dispersed in PS films displayed a pretty much slower reduction in fluorescence emission, i.e., showing 50% decreasing only after 180 s of exposure without levelling off before 4 minutes [28]. This outstanding result was addressed to the prompt and simultaneous response of all the thickness of the films provided by the well isolated and homogeneously distributed covalently linked FMR moieties. In the covalent approach, the external contamination experienced by the polymer matrix is readily perceived by the julolidine-based FMRs, which enable a fast fluorescence response. Conversely, physically dispersed FMR molecules may form clustered supramolecular assemblies, which might adversely affect the fluorescence quenching rate. In fact, blend PS films containing dispersed julolidine-based FMR with a thickness lower than 20–30 µm were not found suitable for sensing experiments due to poor film homogeneity.

P(STY-co-JCBF)(0.34) films were then exposed to hexane vapours, i.e., a solvent with different vapour pressures and Flory–Huggins interaction parameter *χ* (Table 3). The parameter *χ* is defined to give an effective measure of the interaction between the polymer and the solvent. Notably, small *χ* values are characteristic of well-interacting solvent-polymer pairs [41] and, therefore, associated to stronger fluorescence decreasing.

**Figure 5:**
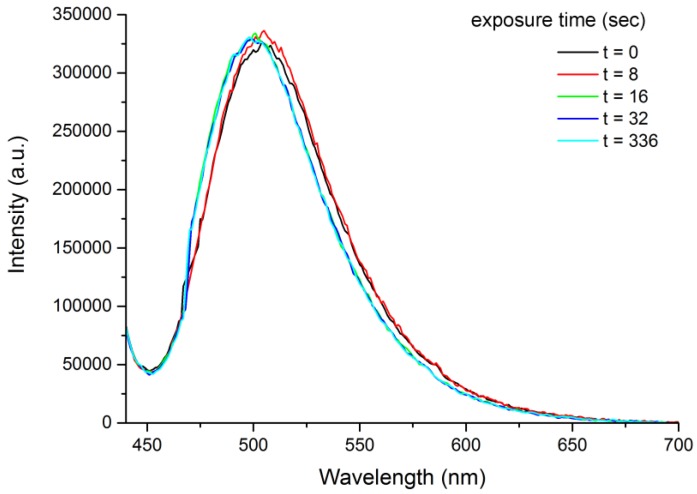
Progressive changes in the fluorescence emission (*λ_exc._* = 410 nm) of P(STY-co-JCBF)(0.34) film as a function of the exposure to hexane vapours. The spectra were collected for a total time of 5–6 min.

As a matter of fact, the exposure of P(STY-co-JCBF)(0.34) film to hexane vapours did not cause any significant variation of their emission intensity (Figure 5).

**Figure 6:**
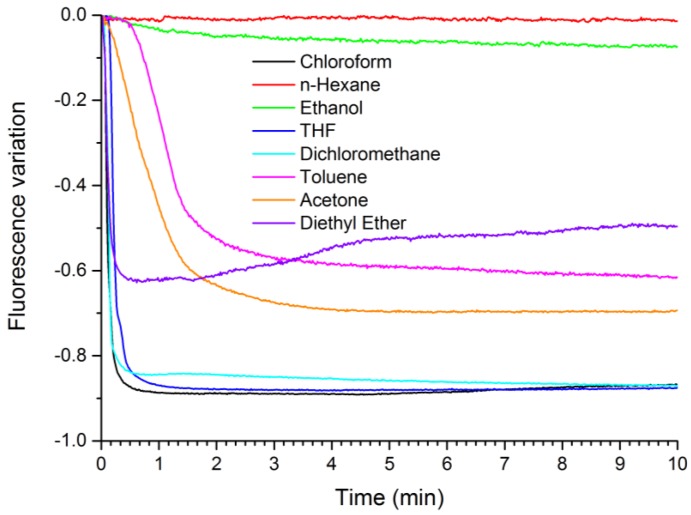
Variation of the fluorescence maximum intensity (*λ_exc_*_._ = 410 nm) of P(STY-co-JCBF)(0.34) films with exposure time to all VOCs investigated.

Significant fluorescence variation of P(STY-co-JCAEM)(0.34) films actually occurred when highly interacting solvents were utilized as VOCs (Figure 6). Notably, Et_2_O, THF, chloroform and dichloromethane produced the fastest decreasing rate (Figure 6) thanks to the favourable combination of χ and vapour pressure. In the case of Et_2_O, the fluorescence drop was not completed since the intensity of the emission started recovering after a certain exposure time. This phenomenon was tentatively addressed to the very high vapour pressure and reduced size of the Et_2_O molecule. These factors caused a rapid adsorption, diffusion and macromolecules rearrangement into a less swellable system that acts as a negative feedback for the vapochromic behaviour. This phenomenon possibly occurs for all the VOCs investigated, most probably shifted at longer exposure times.

The vapochromic response differ significantly for acetone (*χ* = 0.81–0.94) and toluene (*χ* = 0.42–0.31). While for acetone the slower vapochromism was addressed to its higher *χ* value (i.e., *χ* = 0.81–0.94 for acetone against 0.17–0.05 for Et_2_O), for toluene the fluorescence variation was mostly delayed due to its lower vapour pressure (i.e., 2.9 kPa for toluene against 71.7 kPa for Et_2_O, at 25 °C). Moreover, the fluorescence intensity resulted barely affected by hexane (*χ* = 1.49–1.14) and ethanol (*χ* = 2.44) vapours, i.e., for VOCs with limited affinity with the polymer matrix.

It is worth noting that the experimental setup did not affect P(STY-co-JCBF) film size and aspect after the time interval investigated, i.e., within 20 min of VOCs exposure. Actually, solvent can be effectively desorbed at 20 °C and 1 Atm, allowing complete recovery of the film emission after one day. This allowed also the complete reversibility (and reuse) of the designed vapochromic system as evidenced by the good reproducibility of the vapochromic response during the second cycle of chloroform vapours exposure (Figure S6).

**Figure 7:**
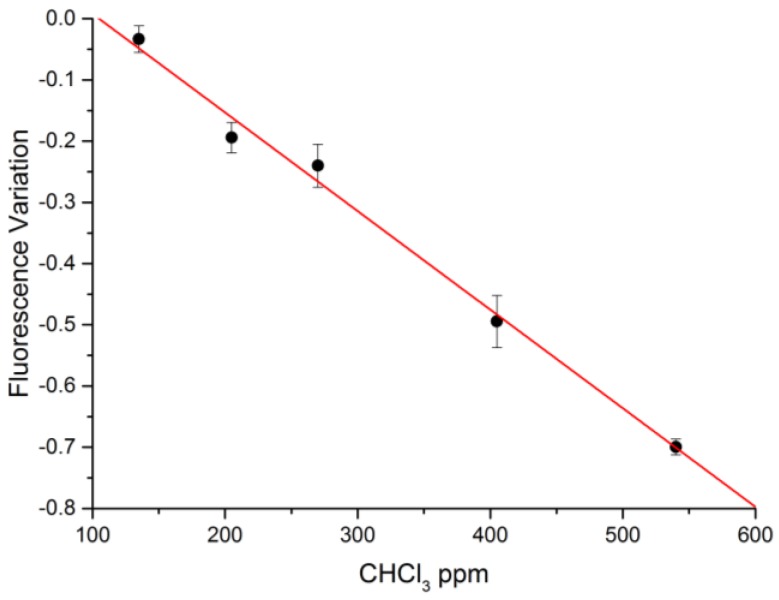
Fluorescence maximum variation as a function of chloroform concentration (ppm, expressed as vol ratio of vaporized chloroform in the measuring chamber).

Lastly, we investigated the sensibility threshold of the P(STY-co-JCBF) films, by placing progressive amounts of chloroform within the measurement box (Figure S7 and Figure 7). The results reported in Figure 7 show that the system started to be optically responsive for concentration of chloroform less than 150 ppm, a threshold that is fairly far from that required in sensors applications.

Nevertheless, the linear relationship between the fluorescence maximum variation and chloroform concentration renders the designed vapochromic polymer films as one of the most promising in the development of modern optical sensors to VOCs.

## 3. Experimental Section

### 3.1. Materials and Methods

All the chemical agents and solvents were obtained from commercial sources and used as received. Phosphorous oxychloride (Sigma-Aldrich, Milan, Italy), was purified by distillation at reduced pressure.AIBN was recrystallized from acetone. *N,N*-dimethylformamide (DMF), THF and toluene were refluxed over CaH_2_ for 2 h and distilled under nitrogen. Styrene was distilled at reduced pressure in presence of butylated hydroxytoluene (BHT, Aldrich) as polymerization inhibitor. Spectroscopy grade solvents (Carlo Erba , Milan, Italy or Sigma-Aldrich, Milan, Italy) were utilized without further purification. 

Fourier transform infrared (FT-IR) spectra were recorded on a Spectrum GX FT-IR (Perkin-Elmer, USA) at room temperature, on pellets made from grinding mixtures of anhydrous KBr with ~1% *w*/*w* of the solid product, and pressing the mixture. Electron ionization mass (EI-MS) spectra were recorded at 70 eV by gas-liquid chromatography and mass spectrometry (GLC-MS), performed on an Agilent 6890N gas-chromatograph interfaced with Agilent 5973N mass detector (Agilent, Santa Clara, CA, USA). NMR spectra (at 400 MHz (^1^H) and 100 MHz (^13^C)) were recorded with a Bruker Advance DRX 400 spectrometer (Bruker, Billerica, MA, USA) at room temperature and were referenced to the residual protons of deuterated solvents.

Gel permeation chromatography (GPC) was used to determine molecular weights and molecular weight dispersion (*M_w_*/*M_n_*) of polymer samples with respect to polystyrene standards. GPC measurements were performed in CHCl_3_ as solvent on a four-channel pump PU-2089 Plus chromatograph (Jasco, Easton, MD, USA) equipped with a Jasco RI 2031 Plus refractometer and a multichannel Jasco UV-2077 Plus UV-Vis detector set at 252 and 360 nm. The flow rate was 1 mL·min^−1^ at a temperature of 30 °C held through a Jasco CO 2063 Plus Column Thermostat. A series composed by two PLgel™ MIXED D columns and a PLgel™ precolumn (Polymer Laboratories, Church Stretton, UK) packed with polystyrene-divinylbenzene was used to perform the analysis (linearity range 100 Da–400 kDa).

Thermogravimetric (TG) analyses were carried out by means of a TGA/SDTA 851 apparatus (Mettler Toledo, Columbus, OH, USA). Samples were heated from 25 to 700 °C at 10 °C·min^−1^ under a nitrogen flow. Differential Scanning Calorimetry (DSC) thermograms were recorded under nitrogen atmosphere by a Mettler Toledo DSC 922e Module Stare apparatus equipped with a liquid nitrogen cooling system. Samples were heated from 25 to 180 °C at 10 °C·min^−1^, then cooled to 0 °C at the same speed. The heating was repeated in the same conditions after 3 min of annealing at 0 °C.

Absorption spectra were recorded at room temperature on a Perkin-Elmer Lambda 650 spectrometer. Fluorescence spectra were measured at room temperature on a Fluorolog^®^-3 spectrofluorometer (Horiba Jobin-Yvon, Palaiseau, France) equipped with a 450 W xenon arc lamp, double-grating excitation and single-grating emission monochromators.

The fluorescence quantum yield (*Φ*) in methanol/glycerol mixtures was determined at room temperature relative to perylene ($\Phi_{f}^{s}$ = 0.92 in EtOH) [35] according to the relation:

(3) $$\Phi =\Phi_{ST}\frac{\int_{0}^{\infty }I\left(\nu \right)d\nu }{\int_{0}^{\infty }I_{ST}\left(\nu \right)d\nu }\frac{\left(1-10^{-A_{ST}}\right)}{\left(1-10^{-A}\right)}\frac{n^{2}}{n_{ST}^{2}}$$

where the subscripts *ST* are referred to standard, the integrals $\int_{0}^{\infty }I\left(\nu \right)d\nu$ and $\int_{0}^{\infty }I_{ST}\left(\nu \right)d\nu$ are the areas under the emission curves of the investigated compound and standard, *A* and *A_ST_* are the absorbances of the investigated compound and standard at the excitation wavelength (410 nm), n and $n_{ST}$ are the refractive index of the solvents, i.e., 1.332 for methanol, 1.474 for glycerol. The refractive index of MeOH/Glycerol mixtures was predicted by the Arago-Biot additive rule [44]:

(4) $$n_{mix}=\phi_{1}n_{1}+\phi_{2}n_{2}$$

The chemical composition of copolymers was evaluated by UV–Vis spectroscopy by means of a calibration curve obtained form 5 × 10^−7^–1 × 10^−5^ CHCl_3_ solutions of JCBF. 

Emission spectra (λ_exc_ 410 nm) of polymer films were recorded on the same spectrofluorometer in the dark by using a F-3000 Fibre Optic Mount apparatus (Horiba Jobin-Yvon, Palaiseau, France) coupled with optical fibre bundles. Light generated from the excitation spectrometer is directly focused on the sample using optical fibre bundles. Emission from the sample is then directed back through the bundle into the collection port of the sample compartment. The emission response of the films was tested by exposing the sample held by a steel tripod in a 50-mL beaker closed by a pierced aluminium foil lid (Scheme 1), to 20 mL of various organic solvents of different vapour pressure and PS-solvent Flory–Huggins interaction parameter *χ* (Table 3), at 25 °C and atmospheric pressure.

### 3.2. Synthesis of 9-Formyljulolidine (FJUL)

The synthesis of 9-formyljulolidine was carried out modifying a reported procedure [45,46]. In brief, phosphorous oxychloride (1.1 mL, 11.55 mmol) was added dropwise to *N*,*N*-dimethyl-formamide (2 mL, 25.85 mmol) at 0 °C. A solution of julolidine (2.0015 g, 11.55 mmol) in DMF (3.5 mL, 45.24 mmol) was then added and the mixture was stirred at 90 °C for 4.5 h. The solution was allowed to cool at room temperature (rt) and neutralized to pH 6–8 by the addition of a saturated sodium acetate solution (~30 mL). After stirring overnight at rt, a greenish-yellow solid precipitate was recovered via filtration, washed with water (30 mL) and dried under high vacuum. The crude product was purified through column chromatography on silica gel using ethyl acetate/CHCl_3_ (70/30 *v*/*v*) as eluent mixture. 1.65 g of FJUL were recovered (71% yield). FT-IR (KBr, cm^−1^): 2758, 1651, 1594, 1527, 1321. ^1^H-NMR (CDCl_3_): δ (ppm) = 9.6 (s, 1H, CHO), 7.3 (s, 2H, aromatic), 3.3 (t, *J* = 5.8 Hz, 4H, NCH_2_), 2.7 (t, *J* = 6.3 Hz, 4H, NCH_2_CH_2_CH_2_), 1.9 (m, 4H, NCH_2_CH_2_). ^13^C-NMR (CDCl_3_): δ (ppm) = 190.1 (-CHO), 147.9 (-N-C(-C-)=C-), 129.5 (-C(=C)-CH=C(-C)-CH=), 124.0 (-CH-(CH=)C-CHO), 120.33 (-CH_2_-C(=C-)-CH(=C)), 50.0 (-N(-CH_2_)-), 27.7 (-N(-CH_2_-CH_2_-CH_2_-)-), 21.3 (-N(-CH_2_-CH_2_-CH_2_-)-). EI-MS *m*/z (%): 201 (100, M^+^).

### 3.3. Synthesis of 2-[4-Vinyl(1,1′-biphenyl)-4′-yl]-acetonitrile (CBF)

2-(4-Bromophenyl)acetonitrile (0.5562 g, 2.84 mmol), 4-vinylphenylboronic acid (0.4469 g, 3.02 mmol) and palladium-tetrakis(triphenylphosphine) (0.045 g, 0.0389 mmol) were dissolved in a mixture of toluene (20 mL), Aliquat 336 (0.16 g, 0.5 mmol) and 2 M aqueous potassium carbonate solution (10 mL). The mixture was stirred at room temperature for 30 min under N_2_ gas and then heated to 90 °C for 24 h. After that the mixture was poured into water and extracted three times with ethyl acetate. The organic layer was dried over anhydrous magnesium sulfate. The crude product was purified through column chromatography on silica gel using petroleum ether/chloroform (3:7) as eluent to a solid white powder. 0.404 g of CBF was recovered (65% yield). FT-IR (KBr, cm^−1^): 2908, 2862, 2250, 1600, 1498, 900, 800. ^1^H-NMR (CDCl_3_): δ (ppm) 7.61 (d, *J* = 7.5 Hz, 2H), 7.57 (d, *J* = 7.5 Hz, 2H), 7.44 (d, *J* = 6.9 Hz 2H), 6.78 (dd, *J* = 7.9, 2.1 Hz, 1H), 5.85 (d, *J* = 16 Hz, 1H), 5.33 (d, *J* = 10 Hz, 1H), 3.82 (s, 2H). ^13^C-NMR (CDCl_3_): δ (ppm) = 140.6 ((-CH-(CH=)C-C(=CH)-CH)), 139.6 ((-CH-(CH=)C-C(=CH)-CH)),), 137.1 ((-CH-(CH=)C-CH), 136.3 ((-CH-(CH=)-C-CH=CH_2_), 128.9 (-CH_2_-C-(=CH)-CH)), 128.4 (-CH_2_-C-(=CH)-CH)), 126.8 (NC-CH_2_-C-(=CH)-CH)), 117.8 (NC-CH_2_-C-(=CH)-CH)), 114.2 (((-CH-(CH=)-C-CH=CH_2_)), 23.34 NC-CH_2_-C-(=CH)-CH)).

### 3.4. Synthesis of 2-[4-Vinyl(1,1′-biphenyl)-4′-yl]-cyanovinyljulolidine (JCBF)

tBuOK (190 mg, 1.70 mmol) was added to a solution of FJUL (0.2538 g, 1.2572 mmol) and CBF (0.3452 mL, 1.574 mmol) in anhydrous THF (10 mL). The mixture was kept under stirring at rt for 2 h. The solvent was then removed under reduced pressure. The crude product was purified through column chromatography on silica gel using chloroform as eluent to a solid white powder. 0.250 g of JCBF was recovered (50% yield). FT-IR (KBr, cm^−1^): 2928, 2854, 2210, 1593, 1523, 1311, 1136. ^1^H-NMR (CDCl_3_): δ (ppm) = 7.75–7.48 (m, 10H, aromatics), 6.79 (dd, *J* = 7.9, 2.1 Hz, 1H, -CH=CH_2_), 5.83 (d, *J* = 16 Hz, 1H, -CH=CH_2_-), 5.31 (d, *J* = 10 Hz, 1H, -CH=CH_2_-), 3.31 (t, *J* = 5.8 Hz, 4H, -N-CH_2_-), 2.82 (t, *J* = 6.3 Hz, 4H, -N-CH_2_-CH_2_-CH_2_), 2.03 (m, 4H, -N-CH_2_-CH_2_). ^13^C-NMR (CDCl_3_): δ (ppm) = 145.1 (CH-(CH=)C-CH-C), 142.3 (-N-C(-C)=C-), 139.8 ((-CH-(CH=)C-C(=CH)-CH)), 139.72 ((-CH-(CH=)C-C(=CH)-CH))), 136.84 ((-CH-(CH=)C-CH), 136.4 ((-CH-(CH=)-C-CH=CH_2_), 129.1 (-CH_2_-C-(=CH)-CH)), 125.7 (NC-CH_2_-C-(=CH)-CH)), 120.8–120.6 (-CH_2_-C(=C-)CH(=C)), 119.7 (NC-C-C-(=CH)-CH)), 114.1 (((-CH-(CH=)-C-CH=CH_2_)), 102.28 (NC-C-C-(=CH)-CH)), 49.9 (-N(-CH_2_)-), 27.7 (-N(-CH_2_-CH2-CH2-)-), 21.5 (-N(-CH_2_-CH2-CH2-)-). EI-MS *m*/z (%): 402 (100, M^+^).

### 3.5. Synthesis of Random Copolymers P(STY-co-JCBF)(m)

We describe in detail the typical preparation for the P(STY-co-JCAEM)(0.34). A solution of JCBF (0.0104 g, 0.025 mmol), styrene (0.55 mL, 4.800 mmol) and AIBN (0.0060 g, 0.037 mmol) in anhydrous toluene (5.2 mL) was introduced into a dry reaction tube with a Rotaflow Polytetrafluoroethylene (PTFE) tap under nitrogen. After three freeze-pump-thaw cycles, the tube was sealed under vacuum through the PTFE screw at the top and the polymerization was let to proceed at 60 °C for 7 days. After cooling to rt, the polymer was then recovered by precipitation into methanol. The polymer was centrifuged and the supernatants collected and then dried at reduced pressure. The polymer was finally purified by repeated precipitations from dichloromethane solutions into methanol. Yield 40%; *M_n_* = 11,000 g mol^−1^ (by GPC). FT-IR (KBr, cm^−1^): 3100–3000, 2925, 2852, 2204, 1942–1745, 1600, 1493, 900, 800. ^1^H-NMR (CDCl_3_): δ (ppm) = 7.20 (aromatic), 6.60 (aromatic), 1.86 (broad, 1H, -CH_2_-CH(-C_6_H_5_)-), 1.45 (broad, 2H, -CH_2_-CH(-Ph)-) typical of polystyrene and 3.20 (4H, -N-CH_2_-), 2.82 (4H, -N-CH_2_-CH_2_-CH_2_), characteristic of JCBF. 

### 3.6. Preparation of P(STY-co-JCBF)(m) Films

Thin polymer films (4 µm) of P(STY-co-JCBF)(m) were obtained by spin-coating on glass substrate. A 2.4 × 2.4 cm glass cover slip was cleaned and then placed on the vacuum chuck hold-down of a WS-400B-6NPP-LITE (Laurell Technologies Corp., North Wales, PA, USA) spin-coater. A viscous solution of the copolymer (5 mg) in CHCl_3_ (40 µL) was placed in the centre of the glass, and the coating was performed at a 750 rpm for 22 s, with an acceleration index of 004 (~448 rpm·s^−1^). The obtained films were allowed to slowly dry at rt for 24 h before any measurement. Film thickness was measured with a CM1S dial indicator (Borletti, Milan, Italy ) with ruby movement bearing.

## 4. Conclusions

We have demonstrated that 2-[4-vinyl(1,1′-biphenyl)-4′-yl]-cyanovinyljulolidine (JCBF), a new julolidine-based FMR, once copolymerized with styrene, endows the resulting P(STY-co-JCBF)(m), with *m%* = 0.10–1.00 thin films with significant vapochromic features. Thin films of styrene copolymers disclosed viscosity-dependent fluorescence once exposed to volatile and well interacting VOCs/polymer pairs. The pronounced drop in their fluorescence was addressed to solvent-induced changes in the local viscosity of the polymer matrix, and appeared about 18 times faster than that of the most efficient julolidine-based FMR dispersed in PS films. Moreover, the films’ size and morphology resulted unaffected by VOCs exposure and the vapochromic response appeared also reproducible. By contrast, the films fluorescence appeared unaltered when scarcely interacting VOCs like ethanol and hexane were utilized. The sensitivity threshold was found to be less than 150 ppm, but the linear relationship between the fluorescence maximum variation and VOC concentration supports the application of julolidine-enriched styrene copolymers as modern vapochromic plastic sensors.

## Figures and Tables

**Table 1:** Characteristics of the *P(STY-co-JCBF)* copolymers.

Copolymer	JCBF (alimentation wt %)	*m* (mol. %)	*M_n_* (g/mol)	*M_w_*/*M_n_*	*T_g_* (°C)	*T_d_* (°C)
P(STY-co-JCBF)(0.10)	0.39	0.10	13,800	1.6	103	415
P(STY-co-JCBF)(0.34)	0.98	0.34	11,000	1.6	102	420
P(STY-co-JCBF)(0.90)	3.41	0.90	10,900	1.5	105	430
P(STY-co-JCBF)(1.04)	3.90	1.04	12,200	1.5	108	430

M_w_: weight average molecular weight; M_n_: number average molecular weight; T_g_: glass transition temperature; T_d_: degradation temperature

**Table 2:** Optical features of 5 × 10^−6^ M JCBF solutions.

Solvent	*A_max_* (nm)	*Em_m_*_ax_ (nm)	*ε* (F/m) ^a^	*η* (mPas) ^b^	*Φ_f_* ^d^
methanol	407	500	33	0.6 (0.6) ^c^	1.26 × 10^−3^
methanol/glycerol 90:10 *v*/*v*	411	505	34.4	1.8	1.56 × 10^−3^
methanol/glycerol 80:20 *v*/*v*	415	506	35.6	4.8	1.79 × 10^−3^
methanol/glycerol 70:30 *v*/*v*	426	508	36.7	7.7	2.36 × 10^−3^
methanol/glycerol 60:40 *v*/*v*	427	510	37.6	13	3.15 × 10^−3^
methanol/glycerol 50:50 *v*/*v*	428	512	38.6	28 (30) ^c^	4.18 × 10^−3^
methanol/glycerol 40:60 *v*/*v*	428	515	39.3	58 (55) ^c^	6.26 × 10^−3^
methanol/glycerol 30:70 *v*/*v*	435	519	40.1	100 (120) ^c^	1.06 × 10^−2^
methanol/glycerol 20:80 *v*/*v*	440	521	40.8	250 (250) ^c^	1.89 × 10^−2^
methanol/glycerol 10:90 *v*/*v*	465	525	41.4	630 (620) ^c^	3.73 × 10^−2^

^a^ Dielectric constants [32], ^b^ viscosity at 20 °C [33] and at ^c^ 22 °C [34]; ^d^ Fluorescence (*λ_exc_*_._ = 410 nm) quantum yield (*Φ_f_*) determined at room temperature relative to perylene ($\Phi_{f}^{s}$ = 0.92 in EtOH) [35].

**Table 3:** Vapour pressure of different solvents at 25 °C, [42] PS-solvent Flory-Huggins interaction parameter *χ*, [43] for the investigated volatile organic compounds (VOCs).

Solvent	Vapour Pressure (kPa)	*χ*
hexane	13	1.49–1.14
toluene	2.9	0.42–0.31
THF	21.6	0.16–0.70
CHCl_3_	21.3	0.52–0.17
CH_2_Cl_2_	58.2	0.78–0.71
Et_2_O	71.7	0.17–0.05
acetone	30.8	0.81–0.94
EtOH	5.7	2.44

## Supplementary Materials

The following are available online: Figure S1: UV-Vis absorption and emission (λ_exc_ exc. = 410 nm) of 1x10^-5^ M JCBF in chloroform, Figure S2: Förster-Hoffmann relationship of 1x10^-5^ M JCBF solutions in methanol/glycerol mixtures with different glycerol volume contents, Figure S3: UV-Vis absorption and emission (λ_exc._ = 410 nm) of 0.5 mg/mL P(STY-co-JCBF)(0.34) in chloroform, Figure S4: (a) UV-Vis absorption and (b) emission (λ_exc._ = 410 nm) of 0.5 mg/mL P(STY-co-JCBF) in chloroform, Figure S5: (a) UV-Vis absorption and (b) emission (λ_exc._ = 410 nm) of P(STY-co-JCBF) thin films, Figure S6: Variation of the fluorescence maximum intensity of P(STY-co-JCBF)(0.34) film as a function of successive cycles of chloroform exposure, Figure S7: Fluorescence variation for all the P(STY-co-JCBF)(m) films as a function of progressive concentration of chloroform (ppm). See Figure 6 for the exact concentration.
