# Supplementary material for: Fluorescent Polystyrene Films for the Detection of Volatile Organic Compounds Using the Twisted Intramolecular Charge Transfer Mechanism
Source: Molecules. 2017 Aug 6;22(8):1306. doi: 10.3390/molecules22081306 (PMC6152380; doi:10.3390/molecules22081306)
Supplement: Supplementary file 1 [file molecules-22-01306-s001.pdf]

Electronic supplementary information (ESI) of the manuscript entitled "Fluorescent polystyrene films operating in TICT mechanism for the detection of volatile organic compounds by Mirko Borelli, Giuseppe Iasilli, Pierpaolo Minei, Andrea Pucci

Corresponding author:
Andrea Pucci, Dipartimento di Chimica e Chimica Industriale, Università di Pisa, Pisa, Italy;
e-mail: andrea.pucci@unipi.it

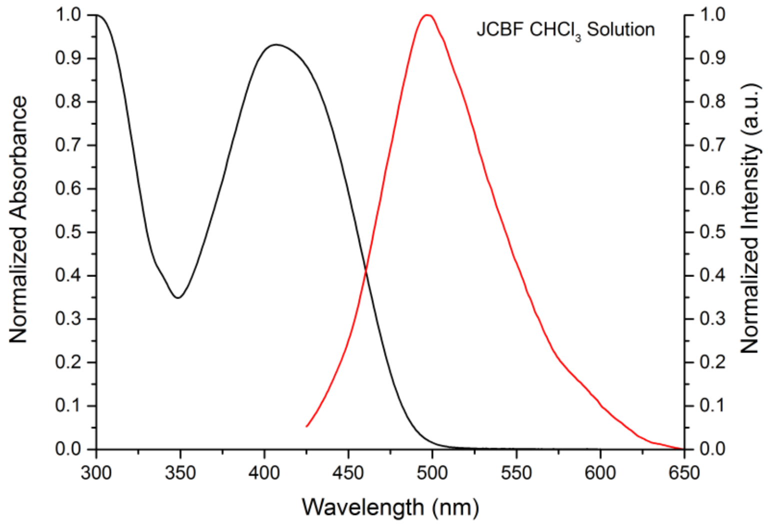

Figure S1. UV-Vis absorption and emission ($\lambda_{exc.}$ = 410 nm) of $1 \cdot 10^{-5}$ M JCBF in chloroform

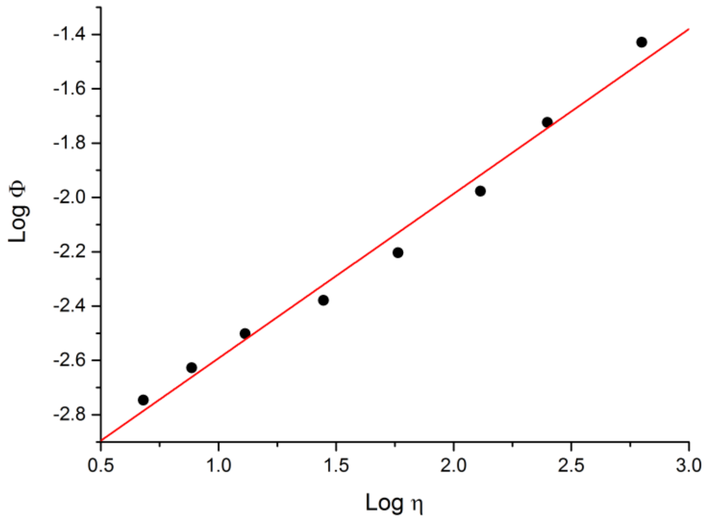

Figure S2. Förster-Hoffmann relationship of $1 \cdot 10^{-5}$ M JCBF solutions in methanol/glycerol mixtures with different glycerol volume contents

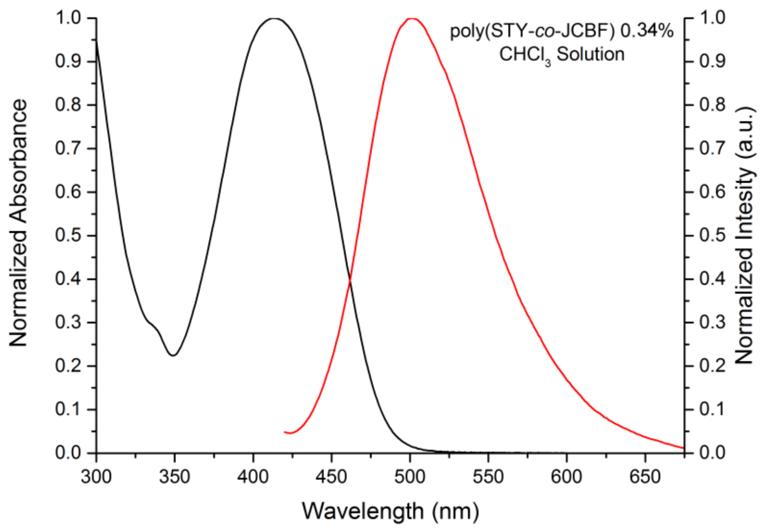

Figure S3. UV-Vis absorption and emission ($\lambda_{exc.}$ = 410 nm) of 0.5 mg/mL P(STY-co-JCBF)(0.34) in chloroform

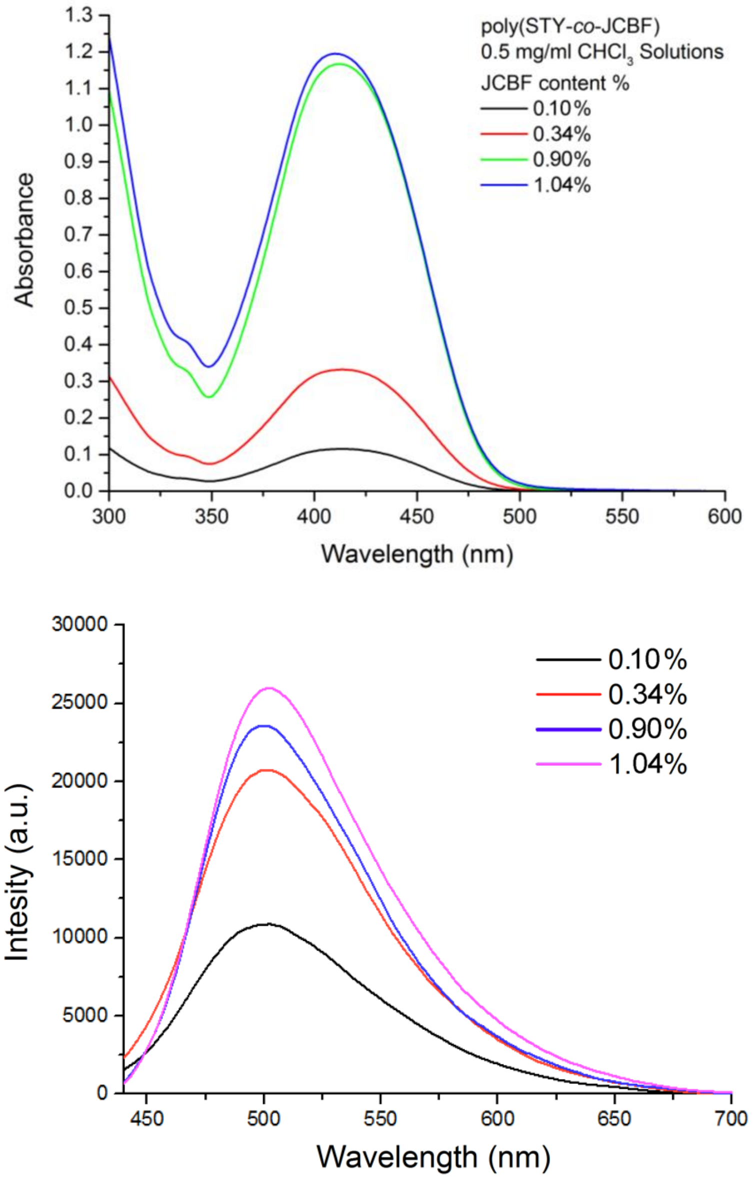

(a)

(b)

Figure S4. (a) UV-Vis absorption and (b) emission ($\lambda_{exc.}$ = 410 nm) of 0.5 mg/mL P(STY-co-JCBF) in chloroform

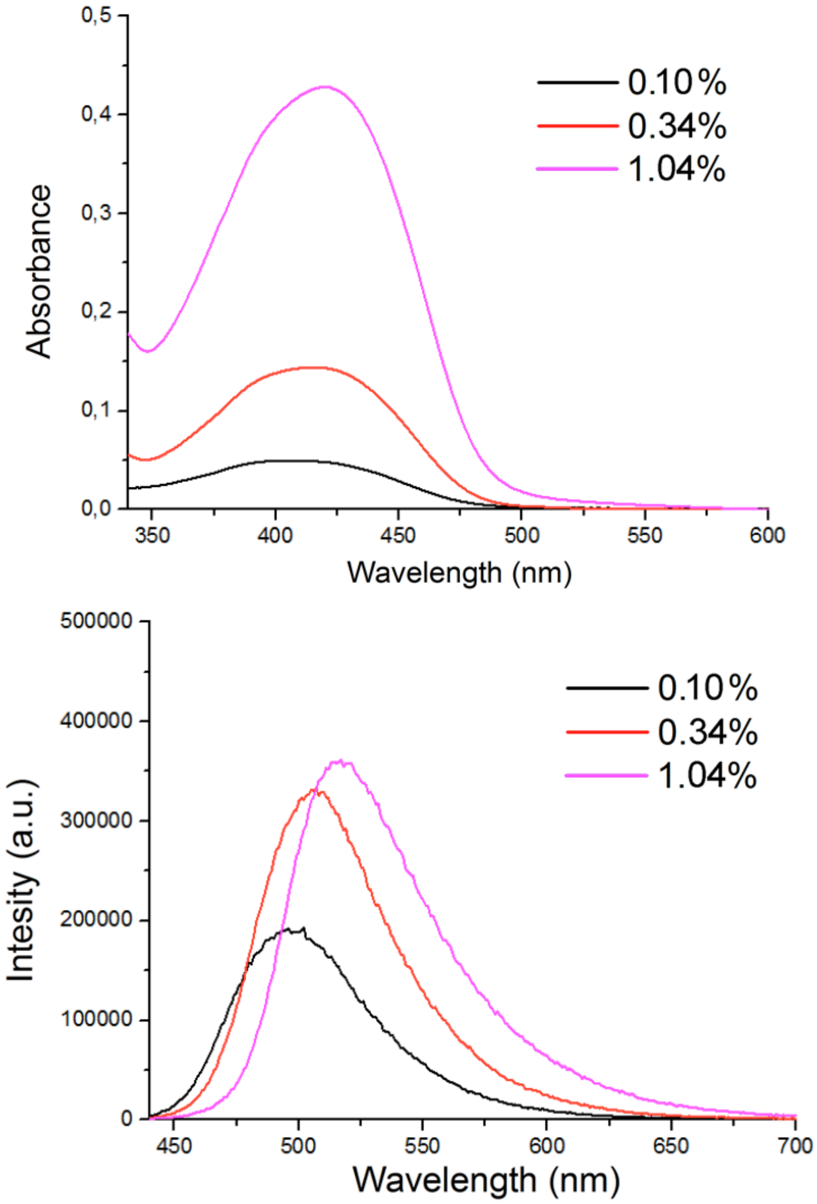

(a)

(b)

Figure S5. (a) UV-Vis absorption and (b) emission ($\lambda_{exc.}$ = 410 nm) of P(STY-co-JCBF) thin films

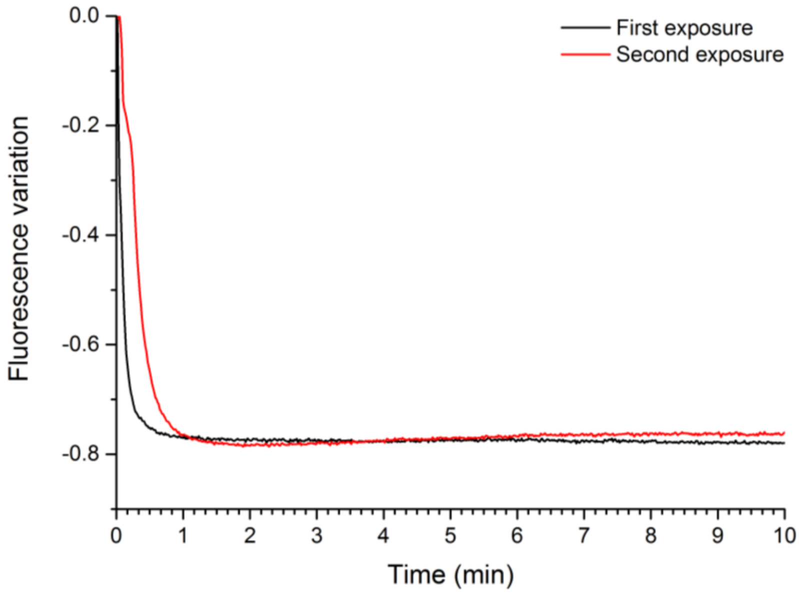

Figure S6. Variation of the fluorescence maximum intensity of P(STY-co-JCBF)(0.34) film as a function of successive cycles of chloroform exposure

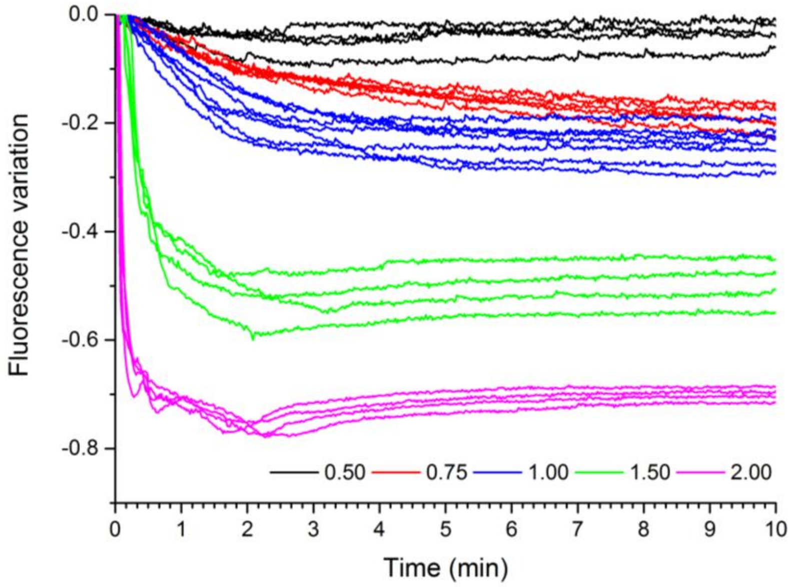

Figure S7. Fluorescence variation for all the P(STY-co-JCBF)(m) films as a function of progressive concentration of chloroform (ppm). See figure 6 for the exact concentration.
